# Comparison of in-person versus virtual ultrasound instruction for pediatric residents

**DOI:** 10.1186/s12909-024-05196-6

**Published:** 2024-02-27

**Authors:** Jason T. Gillon, E. Liang Liu, Valerie Dutreuil, Stephanie G. Cohen, Lekha A. Shah

**Affiliations:** 1 Department of Pediatrics, LSU Health New Orleans School of Medicine, 200 Henry Clay Ave, 70118 New Orleans, LA USA; 2 Children’s Hospital New Orleans LCMC Health, New Orleans, USA; 3 Department of Pediatrics, Division of Pediatric Emergency Medicine, Emory University School of Medicine, Atlanta, GA USA; 4 Children’s Healthcare of Atlanta, Atlanta, GA USA; 5 Department of Emergency Medicine, Emory University School of Medicine, Atlanta, GA USA; 6 Department of Pediatrics, Pediatric Biostatistics Core, Emory University School of Medicine, Atlanta, GA USA

**Keywords:** Ultrasound, Medical education, Virtual, Pediatrics

## Abstract

**Purpose:**

Point-of-care ultrasound (POCUS) instruction is prevalent in medical schools but not in pediatric residency programs, even though the majority of pediatric residents desire POCUS instruction. Virtual ultrasound instruction with affordable handheld ultrasound devices may help remedy this deficiency by allowing qualified instructors to circumvent geographic and financial limitations to reach this population. This study sought to determine if virtual ultrasound instruction is an effective alternative to traditional in-person instruction in a cohort of pediatric residents for the extended Focused Assessment with Sonography in Trauma (eFAST) exam.

**Methods:**

Pediatric residents were randomized to receive either in-person or virtual instruction to learn the eFAST exam using a Sonosite Edge (Sonosite, Inc., Bothell, WA) or Butterfly iQ (Butterfly Network, Inc., Guilford, CT), respectively. After the instructional session, the participants completed a timed assessment in which all required images for the eFAST exam were obtained on the same anatomic model. The content and quality of the images were then scored by expert faculty.

**Results:**

There were no significant differences in assessment scores (65.8% and 61.8%, *p* = 0.349) and assessment duration (482.6 s and 432.6 s, *p* = 0.346) between pediatric residents who received in-person instruction and those who received virtual instruction.

**Conclusion:**

Virtual ultrasound instruction appears to be an effective alternative to traditional in-person instruction.

## Background

Point-of-care ultrasound (POCUS) has become a highly utilized tool in emergency departments (EDs) and is a required part of the core curriculum for emergency medicine residencies [1]. There are no established guidelines for teaching POCUS to pediatric residents despite the potential benefits of POCUS use in children in ED and intensive care settings [2]. A survey of pediatric residents found that 85% received no POCUS training even though an equal percentage desired POCUS instruction, with the majority stating it should be required [3]. A survey of pediatric residency associate program directors showed similar results, with the majority believing pediatric residents should be trained in POCUS, although most had no program in place for resident instruction citing a lack of qualified instructors [4].

POCUS instruction may also be limited by machine availability, with most institutions having a limited number of machines available often due to cost [2, 5]. Recently, several models of portable handheld ultrasound devices costing a fraction of traditional ultrasound machines have entered the commercial market [5]. These new devices also allow for remote learning due to their integration with proprietary internet-based platforms and cloud-sharing.

Virtual instruction became a necessity during the COVID-19 pandemic when social distancing mandates precluded traditional bedside education, and virtual ultrasound curriculums were quickly devised and implemented [6, 7]. This provided proof-of-concept for virtual instruction of a manual skill traditionally taught in-person. With lack of qualified instructors cited as the primary barrier to ultrasound instruction for pediatric residents, virtual instruction provides a viable option for instructors to reach a greater number of students with lower associated costs and no need for travel.

This study sought to determine whether the extended Focused Assessment with Sonography in Trauma (eFAST) exam could be effectively taught to a group of pediatric residents virtually compared to traditional in-person ultrasound instruction. The eFAST exam is used in trauma to detect pneumothorax and hemorrhage in the pericardial, peritoneal, or pleural space and is the most commonly taught POCUS application [8]. It consists of views of the right and left upper quadrants of the abdomen, the pelvis, the pericardium, and the bilateral chest. The instruction in this study focused on the procedure to obtain the requisite images of the eFAST exam, understanding that learning to accurately interpret these images takes time and experience in a real clinical setting.

## Methods

### Participants

Eligible participants for this study were pediatric residents in any year of training from two residency programs who were on the pediatric emergency medicine service at a high-volume urban children’s hospital during the study period from November 2020 to June 2021. Participation was voluntary. Prior ultrasound experience was not an exclusion criteria as ultrasound is now incorporated into the curriculum of most medical schools [9]. A single instructor, who was completing an Advanced Emergency Medicine Ultrasonography (AEMUS) fellowship accredited by the Emergency Ultrasound Fellowship Accreditation Council (EUFAC), conducted all of the instructional sessions.

### Equipment

For virtual instruction, the Butterfly iQ (Butterfly Network, Inc., Guilford, CT), a handheld ultrasound system that uses a single transducer for all applications, was used. The transducer is then connected to a smartphone or tablet. For this study, an iPad mini (Apple Inc., Cupertino, CA) was used to balance screen size with portability. Butterfly TeleGuidance (Butterfly Network, Inc., Guilford, CT), is a wireless internet platform that allows for video calls between the operator and another person who can remotely view the scanner’s ultrasound images in real time. The platform also streams video from the camera of the smartphone or tablet allowing display of where and how the operator is holding the transducer. Augmented reality features that overlay the camera feed allow the instructor to provide visual cues for the scanner to maneuver the transducer into the correct position. Additionally, the gain (brightness) and depth of the image can be remotely adjusted by the instructor, and the instructor can draw on the ultrasound image to highlight structures or features of the image they wish to discuss.

For in-person instruction, the Sonosite Edge (Sonosite, Inc., Bothell, WA), was used. This is a cart-based ultrasound machine with multiple transducer options. For this study, a 5 − 2 MHz curvilinear transducer or a 5 − 1 MHz phased array transducer was used.

### Study design

Pediatric residents who consented to participate in the study were block-randomized in groups of two, three, or four to receive either virtual or in-person instruction. A minimum group number of two was required as the residents scanned each other as models. A pre-study survey to determine prior ultrasound experience and level of interest in POCUS was completed prior to the instructional session. Residents were also asked to watch a narrated video from the Academy of Emergency Ultrasound (AEUS) before the instructional session to provide a conceptual foundation for the eFAST exam [10]. 

Residents in both the virtual and in-person groups were taught individually by the instructor, using a standardized script, scanning protocols to obtain the necessary images for a complete eFAST exam. There was no time limit, and each resident determined when they felt they had received sufficient instruction. Residents took turns receiving one-on-one instruction and serving as a scanning model for their colleagues. The total instructional time was recorded for each resident. Residents receiving virtual instruction connected to the instructor via the TeleGuidance platform. The instructor was unable to directly view or manually assist the residents but remained in the same building to troubleshoot any technological issues if needed.

After the instructional session, residents were asked to schedule an assessment time within seven days and after a minimum of 24 h. During the assessment each resident performed an eFAST exam on a consistent anatomic model using the ultrasound machine they had used for the instructional session. Residents were asked to record cine-loops (videos) of each component of the eFAST exam: right upper quadrant, left upper quadrant, transverse and sagittal views of the pelvis, pericardium, left and right pleura. They were also asked to optimize gain and depth for each cine-loop. The amount of time it took a resident to scan and record each view was documented. After completion of the assessment, residents were asked to complete a post-study survey.

The assessments were reviewed and scored by two physicians fellowship-trained in emergency ultrasound. Each view of the eFAST exam was eligible for a total of five points. The transverse and sagittal views of the pelvis together were worth five points as were the left and right pleurae, equating to a total possible score of 25/25. Of the five points one could earn for each view, one point could be earned if the gain of the image was appropriate. An additional point was given if the image depth was appropriate. The remaining three points were based on the percentage of required anatomy visualized. Zero points were given if no relevant anatomy was visualized, one point for > 0% but < 50%, two points for ≥ 50% but < 100%, and a full three points if all relevant anatomy was visualized. Discrepant scores were arbitrated by a third POCUS-trained physician with greater than ten years of experience.

### Data Analysis

SAS v. 9.4 (SAS Institute, Inc., Cary, NC) was used for all statistical analyses. Demographic information and study variables are summarized in Table 1. The outcome variables of assessment scores and study duration are summarized in Table 2. The groups were compared using two-sample *t*-test for parametric data and Wilcoxon rank-sum test for non-parametric data, with statistical significance determined at the 0.05 threshold. Interrater agreement between the physicians scoring the assessments was determined by Cohen’s kappa coefficient (*κ*).

**Table 1:** Comparison of study characteristics between in-person and virtual groups

Variable	Level	In-Person (N = 24)	Virtual (N = 26)	*P*-value
Year of Training	PGY-1	13 (54.2%)	7 (27%)	0.064
	PGY-2	6 (25.0%)	9 (34.6%)	
	PGY-3	5 (20.8%)	10 (38.5%)	
Prior ultrasound experience	*I have never used ultrasound*	1 (4.2%)	2 (7.7%)	0.846
	*I have used it only once*	4 (16.7%)	3 (11.5%)	
	*I have used it a few times*	15 (62.5%)	18 (69.2%)	
	*I have used it several times*	4 (16.7%)	3 (11.5%)	
	*I have used it on a regular basis*	--	--	
Prior FAST/eFAST exams	*0*	12 (50.0%)	12 (46.2%)	0.877
	*1–10*	4 (16.7%)	7 (26.9%)	
	*10–25*	7 (29.2%)	7 (26.9%)	
	*25–50*	1 (4.2%)	0 (0)	
	*> 50*	--	--	
Training duration (minutes)		18.5 (4.4) ^a^	19.0 (7.2) ^a^	0.786
Time between training and assessment (hours)		52.5 (34.0, 74.0) ^b^	47.0 (28.0,81.0) ^b^	0.475

^*^All continuous summaries are presented: a = mean (SD), b = median (25th -75th percentiles). Categorical summaries are presented as count and percentages. The parametric *p*-values are calculated by two-sample *t*-test and non-parametric *p*-values are calculated by Wilcoxon sum-rank test

**Table 2:** Comparison of outcome variables between in-person and virtual groups

Variable	Bedside (N = 24)	Remote (N = 26)	*P*-value
Total score (out of 25)	16.5 (3.7) ^a^	15.5 (3.7) ^a^	0.349^a^
Gain subscore (out of 5)	4.0 (2.5, 4.25) ^b^	3.3 (2.5, 4.0) ^b^	0.338^b^
Depth subscore (out of 5)	2.6 (1.3) ^a^	2.2 (1.1) ^a^	0.316^a^
Anatomy subscore (out of 15)	10.6 (2.1) ^a^	10.2 (2.1) ^a^	0.536^a^
Assessment duration (seconds)	482.6 (201.4) ^a^	432.6 (170) ^a^	0.346^a^

^*^All continuous summaries are presented: a = mean (SD), b = median (25th -75th percentiles). The parametric *p*-values are calculated by two-sample *t*-test and non-parametric *p*-values are calculated by Wilcoxon sum-rank test

## Results

A total of 50 pediatric residents enrolled in the study, with 24 randomized to the in-person group and 26 randomized to the virtual group. Both groups had similar degrees of prior ultrasound experience, including with eFAST exams (Table 1). A higher proportion of pediatric interns were randomized to the in-person group while a higher proportion of senior residents were randomized to the virtual group, but this difference did not reach significance (*p* = 0.064). Instructional sessions were completed in approximately 20 min per resident, regardless of method of instruction, with assessments occurring on average two days after the instructional sessions.

There were no significant differences in assessment scores between groups, even when stratified by score component (Table 2). The time to completion of the assessment was also not significantly different between groups, taking approximately seven-to-eight minutes of active scanning time to record cine-loops of all required views. Cohen’s kappa coefficient for the two primary scorers was *κ =* 0.094, indicating only slight agreement. Agreement was highest for depth (*κ =* 0.164) and lowest for anatomy (*κ =* 0.046).

**Fig. 1:**
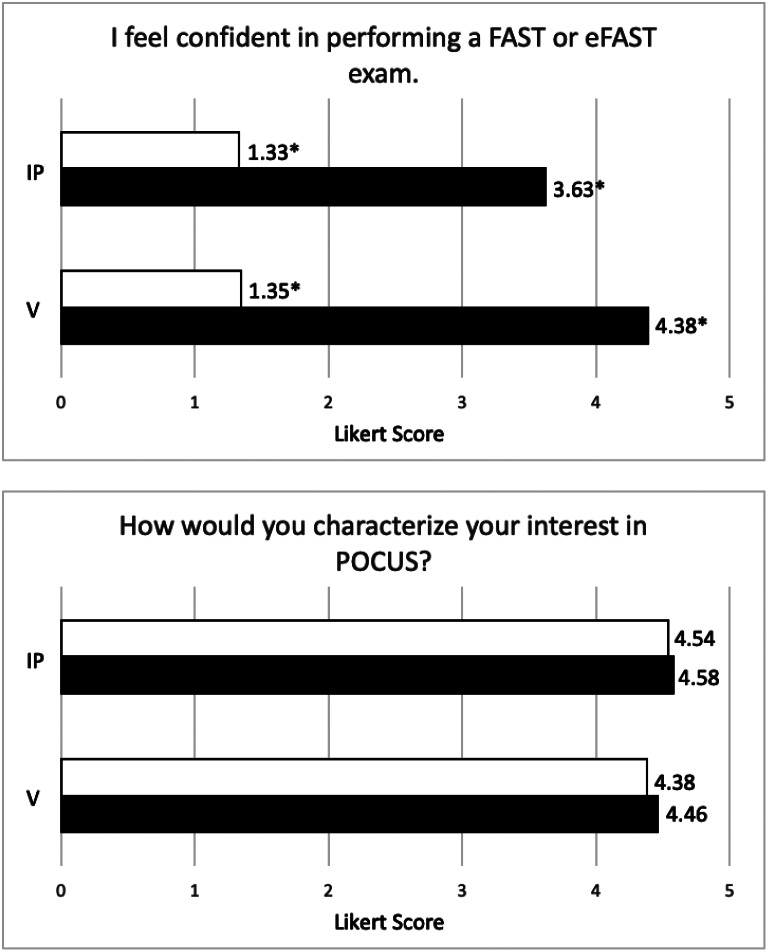
Pre-study (white bars) and post-study (black bars) response averages to survey questions. Answers were given on a Likert scale from 1 (strongly disagree or not at all interested, respectively) to 5 (strongly agree or very interested, respectively). Asterisks (*) denote a significant difference (*p* < 0.05) between pre- and post-study responses

Pre-study interest in POCUS among residents was already very strong, and this remained strong after the study (Fig. 1). Confidence level in performing an eFAST exam significantly increased after the study period. There was a suggestion of higher post-study confidence in the virtual group compared to the in-person group, but this difference did not reach significance (*p* = 0.14).

## Discussion

This study suggests that virtual ultrasound instruction using a wireless internet platform specifically designed for this purpose is an effective instructional modality compared to traditional in-person bedside instruction. Assessment scores were similar between methods of instruction, and residents felt significantly more confident performing an eFAST exam regardless of modality. Importantly, there were no technical issues encountered with the virtual platform, which could be a significant barrier to utility.

Demonstrating the adequacy of virtual POCUS instruction is important as it provides a potentially more accessible option to teach pediatric residents a skill they greatly desire to learn [3]. When soliciting general comments about this study on the post-study survey, many comments mentioned a desire for more instruction, including: “I thought it was really great (…) I just wish there were more sessions,” “Without it, I don’t think I would’ve gotten formal instruction on POCUS,” “I would love to have more of these sessions incorporated into the teachings for the rotation,” and “I hope to see eFAST being taught as a standard part of the pediatric emergency department rotation.” One description of an ultrasound curriculum that was longitudinally integrated into three years of pediatric residency found it to be well-received, with all three years of pediatric residents judging it effective and valuable [11]. While specific POCUS applications are not currently listed as a core procedural competency by the Accreditation Council for Graduate Medical Education (ACGME) for graduate medical education in pediatrics, select pediatric residents may benefit from learning this skill in the context of future career goals and practice settings.

A few studies have already examined virtual ultrasound instruction. Two provide descriptions of programs developed in response to the COVID-19 pandemic. One large program spanning 15 institutions termed A Distance-learning Approach to POCUS Training (ADAPT), which used the same platform as this study, found that the majority of learners felt confident acquiring and interpreting ultrasound images, and the majority of instructors thought the program was effective and recommended its continued use [6]. In another publication, a single institution demonstrated virtual instruction during the pandemic in which learners remotely scanned each other in pairs, which was well received by learners [7]. Though some faculty found that a higher level of expertise was required since they were unable to physically guide the learners’ hand movements, the author makes note that some studies have suggested that verbal and somatosensory feedback during transducer adjustments may be more effective in psychomotor skill development. However, due to the social distancing constraints during the pandemic, these studies could not be compared to traditional instruction methods.

One small study compared assessment scores among anatomy graduate students taking an ultrasound imaging course which was taught in-person before the pandemic and virtually during the pandemic [12]. No significant difference in assessment scores was noted. Another study compared virtual instruction of the FAST exam using a handheld ultrasound device and tele-ultrasound platform (different than the one used for our study) to in-person instruction with the same device [13]. The study population consisted of medical students, and students with prior ultrasound experience were excluded. Like the results of our study, there was no significant difference in assessment scores (which focused on anatomy and not image settings) between the in-person and virtual groups. Since most medical students now have some exposure to ultrasound during medical school, it was neither feasible nor reflective of the current reality to exclude pediatric residents with prior ultrasound experience in our study [9]. Additionally, using larger traditional ultrasound machines for in-person instruction is the standard current model at most institutions and strengthens study generalizability.

While this study highlights the use of portable ultrasound devices and tele-ultrasound platforms for educational purposes, other applications have been recently reported, suggesting growth potential for a technology that may currently seem somewhat novel. In one pilot study, patients with hemophilia were able to scan their joints to assess for hemarthrosis correctly with near-perfect image quality under provider instruction using the Butterfly iQ with TeleGuidance [14]. Another study describes palliative care doctors and nurses in rural settings using handheld point-of-care-ultrasound to assess patients in their homes, with sonographic findings contributing to treatment decisions in half of the cases [15]. Other case reports describe a patient with COVID-19 performing lung-ultrasound daily from home quarantine and a radiologist instructing a military clinician to use ultrasound in a deployed environment, both utilizing the Butterfly TeleGuidance platform [16, 17]. 

This study has several limitations. First, despite requiring residents to view an instructional video of the eFAST exam prior to the instructional session, there was no way to ensure this was done. Second, since the residents scanned each other rather than the same model for convenience reasons, there was anatomic variability. Lastly, since this was a convenience sample of residents with different shift schedules, the exact timing of the assessment after the instructional session could not be controlled.

The interrater agreement in this study was poor, highlighting the subjective nature of what is considered a quality ultrasound image. There is currently no widely adopted validated standard to assess the quality of FAST exam images. Furthermore, it is presently unclear how FAST exam quality correlates with being able to determine the presence of free fluid. One study assessing image quality of aortic scans found no relationship between objective image quality using specific sonographic measurements and subjectively judged image quality, even though abdominal aortic aneurysm screening is a more straightforward study than an eFAST exam [18]. As such, determining more objective quality measures for POCUS studies is an area that requires more attention.

## Conclusions

Virtual ultrasound instruction appears to be an effective alternative to traditional in-person instruction. It may provide a more efficient and cost-effective avenue to provide pediatric residents exposure to a skill they desire to learn. Additional studies with this emerging technology evaluating other ultrasound applications and learner groups are needed.

## Data Availability

The datasets used and/or analyzed during the current study are available from the corresponding author on reasonable request.

## Abbreviations

AEMUS: Advanced Emergency Medicine Ultrasonography
ED: emergency department
eFAST: extended Focused Assessment with Sonography in Trauma
EUFAC: Emergency Ultrasound Fellowship Accreditation Council
POCUS: point-of-care ultrasound
